# High total antioxidant capacity of the porcine seminal plasma (SP-TAC) relates to sperm survival and fertility

**DOI:** 10.1038/srep18538

**Published:** 2015-12-21

**Authors:** Isabel Barranco, Asta Tvarijonaviciute, Cristina Perez-Patiño, Inmaculada Parrilla, Jose J. Ceron, Emilio A. Martinez, Heriberto Rodriguez-Martinez, Jordi Roca

**Affiliations:** 1 Department of Medicine and Animal Surgery, Faculty of Veterinary Science, University of Murcia, Spain; 2 Department of Clinical & Experimental Medicine (IKE), University of Linköping, Sweden

## Abstract

The study attempted to clarify the role of total antioxidant capacity of seminal plasma (SP-TAC) on boar sperm survival and fertility after artificial insemination (AI). SP-TAC differed (*P* < 0.001) among boars (n° = 15) and, to a lesser degree, among ejaculates within male (4 ejaculates/boar). SP-TAC also differed (*P* < 0.001) among ejaculate fractions (43 ejaculates and 3 fractions per ejaculate), of which the sperm-peak portion of the sperm rich ejaculate fraction (SRF) had the highest SP-TAC. SP-TAC was not correlated with sperm quality (motility and viability) or functionality (intracellular ROS generation and lipid peroxidation) of liquid AI-semen samples stored at 17 °C for 72 h (90 AI-samples), but the decline in sperm quality was larger (*P* < 0.05) in ejaculates with low, compared with high SP-TAC (hierarchically grouped). The SP-TAC differences among ejaculate portions agree with sperm cryosurvival rates (14 ejaculates from 7 boars), showing sperm from sperm-peak portion better (*P* < 0.01) post-thaw quality and functionality than those from the entire ejaculate (mainly post-SRF). Boars (n° = 18) with high SP-TAC (hierarchically grouped) had higher (*P* < 0.05) fertility outcomes (5,546 AI-sows) than those with low SP-TAC. Measurement of SP-TAC ought to be a discriminative tool to prognosis fertility in breeding boars.

Artificial insemination (AI) is a highly efficient breeding technology in the pig industry. In addition to their genetic merit, boars included in AI-programs are carefully selected and safeguarded to provide ejaculates with a large number of motile, viable and morphologically normal spermatozoa. Only ejaculates of high sperm quality standard are used for production of AI-semen doses^1^. Despite these rigorous selection criteria, differences among AI-boars are still noteworthy regarding *in vivo* fertility and the ability of their spermatozoa to tolerate preservation^2^,^3^. Therefore, discerning the causes that could explain these differences still remains a challenge^4^.

Ejaculated spermatozoa bathe in seminal plasma (SP), resulting in a direct interaction between most of the chemical SP-components and sperm. It is well known that this interaction contributes to modulate the functional status of boar spermatozoa^5^. Moreover, some SP-components, mainly proteins, show a direct relationship with both *in vivo* fertility and sperm survival post-handling^6^,^7^.

Oxidative stress of the membranes threatens mammalian sperm survival, impairing critical functions such as motility, membrane integrity and even fertilizing ability^8^. Oxidative stress results from an increased generation of reactive oxygen substances (ROS) and/or a decreased available antioxidant defence system^9^. Boar sperm are particularly sensitive to oxidative stress, due to the high proportion of polyunsaturated fatty acids present in the membrane, which can be oxidized leading to lipid peroxidation^10^. Sperm has limited antioxidant defenses to counteract ROS-attack, leaving the SP as source of antioxidants to keep ROS-levels within physiological range, compatible with the functional life of the sperm. Seminal plasma contains many chemical components with antioxidant properties, enzymatic and non-enzymatic^11^, which together determine its total antioxidant capacity (TAC). The determination of SP-TAC has proven to be relevant in humans for fertility assessment because low levels of SP-TAC are associated with infertility and abnormal semen parameters^12^,^13^. So, it is reasonable to assume that quantitative differences in SP-TAC among boar ejaculates would explain differences on sperm capacity to sustain preservation procedures and display best *in vivo* fertilizing ability.

Against this background, the purpose of this study was to evaluate whether there are differences in SP-TAC among breeding boars and whether such differences were related to sperm performance after handling, including cryopreservation, and fertility post-AI. In order to fulfil this goal, 347 semen samples from 177 boars and fertility records of 5,546 AI-sows were considered.

## Material and Methods

### Reagents and media

All of the chemicals used in the experiments were of analytical grade and, unless otherwise stated, purchased from Sigma-Aldrich (St. Louis, MO, USA). Fluorochrome molecules were purchased from Molecular Probes (Europe BV, Leiden, The Netherlands). The basic medium used for sperm extension was Beltsville Thawing Solution (BTS: 205 mM glucose, 20.39 mM Na_3_-C_6_H_5_O_7_, 10.0 mM KCl, 15.01 mM NaHCO_3_, 3.36 mM EDTA; pH 7.2 and 290–300 mOsmol/kg) supplemented with 50 mg/mL of kanamycin sulphate. EDTA-free phosphate buffered saline (PBS: 139 mM NaCl, 2.7 mM KCl, 1.5 mM KH_2_PO_4_, 8.1 mM Na_2_HPO_4_·7 H_2_O; with 0.058 g/L penicillin G and 0.05 g/L streptomycin sulphate; pH 6.8 and 280–300 mOsmol/kg) was used for flow cytometric analysis. Sperm were frozen using a basic freezing medium containing 80% (v/v) Tris-citric acid-glucose extender (111 mM Trizma Base, 31.4 mM monohydrate citric acid, 185 mM glucose) and 20% (v/v) egg yolk, supplemented with 100 μg/mL kanamycin sulphate (pH 7.2 and 295–300 mOsmol/kg).

### Boars, ejaculates and seminal plasma

All procedures that involved animals were performed according to the international guidelines and approved by the Bioethics Committee of Murcia University (research code: 639/2012). Ejaculates, both entire or in portions, were collected, using the gloved-hand method, from healthy and sexually mature boars (2 to 3 yr of age) of different breeds (Landrace, Large White and Pietrain) undergoing regular semen collection for commercial AI (AIM Iberica, Calasparra, Murcia, Spain). All ejaculates used fulfilled the standards of quantity and sperm quality thresholds for the preparation of AI-semen doses (more than 200 × 10^6^ spermatozoa/mL, 70% motile spermatozoa, and 75% of morphologically normal cells).

The SP was harvested after double centrifugation at 1500 g for 10 min (Rotofix 32 A; Hettich Zentrifugen, Tuttlingen, Germany) of 15 mL semen samples. The second supernatant was examined using microscopy to ensure it was sperm-free and thereafter sent in insulated containers (15–17 °C) to the Andrology Laboratory at the Veterinary Teaching Hospital of the University of Murcia. All SP samples arrived within 2–4 h of ejaculate collection. At the laboratory, the SP samples were frozen to −80 °C (Ultra Low Freezer; Haier, Schomberg, Ontario, Canada) and stored until analysed. For analyses, the SP samples were thawed at room temperature.

### Sperm cryopreservation

Semen samples diluted BTS (2:1, v/v) and stored 24 h at 15–17 °C were centrifuged (Megafuge 1.0 R, Heraeus, Hanau, Germany) for 3 min at 2,400 g, and the sperm pellets were frozen using the straw freezing procedure described by Alkmin *et al.*^14^. Briefly, sperm pellets were extended in the basic freezing medium to a concentration of 1.5 × 10^9^ spermatozoa/mL. After cooling to 5 °C for 150 min, the sperm were re-extended in the basic freezing medium supplement with glycerol and Equex (89.5% basic freezing medium + 1.5% Equex STM (v/v) (Nova Chemical Sales, Scituate, MA, USA) and 9% glycerol (v/v); pH 6.2; 1700–1730 mOsmol/kg) to a final concentration of 1.0 × 10^9^ spermatozoa/mL. The spermatozoa were thereafter packed into 0.5 mL polyvinyl chloride (PVC) French straws (Minitüb, Tiefenbach, Germany) and frozen at −40 °C/min using a controlled-rate-freezing machine (IceCube 1810, Minitüb). The straws remained in liquid nitrogen for at least 1 week before thawing, which was performed in a circulating water bath at 37 °C for 20 s. The thawed sperm samples were extended in BTS (1/1, v/v) and incubated at 37 °C for up to 150 min for sperm analyses.

### Determination of Total Antioxidant Capacity (TAC) in seminal plasma samples

The SP-TAC was determined via an automated measurement method developed by Erel^15^. The method used is based on 2, 2′-azinobis-(3-ethylbenzothiazoline-6-sulfonate) decolourization by antioxidants according to their concentration and antioxidant capacity. The colour change is measured as a change in light absorbance at 660 nm. For the process, an automated analyser (AU 400, Olympus, Minneapolis, USA) was used, and the assay was calibrated with 6-hydroxy-2, 5, 7, 8-tetramethylchroman-2-carboxylic acid (Trolox). The TAC value of each SP sample was expressed as an equivalent of the mM-concentration of Trolox solution.

### Assessment of sperm quality and functionality

The spermatozoa were assessed according to quality (total and progressive motility and viability) and functionality (basal intracellular H_2_O_2_ generation and lipid peroxidation) parameters. All of these assessments, except motility, which was evaluated using a computer-assisted sperm analyzer (CASA), were performed using flow cytometry using a BD FACS Canto II flow cytometer (Becton Dickinson & Company, Franklin Lakes, NJ, USA). Hoechst 33342 (H-42) fluorescence (DNA content) was used for identifying sperm events. Acquisition was stopped after 10,000 H-42 positive events.

Sperm motility was objectively evaluated using an ISASV1^®^ CASA (Proiser R + D, Paterna, Spain). For each evaluation, a 5 μL of extended semen (20−30 × 10^6^ spermatozoa/mL in PBS) was placed in a 10 μL-Makler counting chamber (Sefi Medical Instruments, Haifa, Israel) that had been pre-warmed to 38 °C, and six to nine fields, with a minimum of 400 spermatozoa per sample, were analysed. The sperm motility variables recorded were the overall percentage of motile spermatozoa (average path velocity ≥ 20 μm/sec) and the proportion of motile spermatozoa showing rapid and progressive movement (straight line velocity ≥ 40 μm/sec).

For sperm viability assessment, 100 μL of semen (30 × 10^6^ spermatozoa/mL in PBS) were mixed with 3 μL H-42 (0.05 mg/mL in PBS), 2 μL propidium iodide (PI, 0.5 mg/mL in PBS), and 2 μL fluorescein-conjugated peanut agglutinin (PNA-FITC, 100 μg/mL in PBS) and then incubated at 38 °C in the dark for 10 min. Immediately before analysis, 400 μL of PBS were added to each sample. Viable spermatozoa were those exhibiting intact plasma and acrosome membranes (H-42 positive/PI negative and PNA-FITC negative) and were reported as percentages.

The intracellular generation of H_2_O_2_ in viable spermatozoa was measured using 5-(and-6) chloromethyl-20,70-dichlorodihydrofluorescein diacetate acetyl ester (CM-H_2_DCFDA). For each semen sample, 50 μL of diluted spermatozoa (30 × 10^6^ spermatozoa/mL in PBS) were re-extended in 950 μL PBS containing 1.25 μL H-42 (0.05 mg/mL in PBS), 1 μL PI (0.5 mg/mL in PBS) and 1 μL H_2_DCFDA (1 mM in DMSO) to measure basal H_2_O_2_ generation. The samples were incubated at 38 °C in the dark for 30 min prior to flow cytometric analysis. The mean fluorescence intensity was expressed as fluorescence units per 10^6^ viable spermatozoa.

Lipid peroxidation was assessed using BODIPY (581/591) C_11_ following a modified Koppers *et al.*^16^ procedure. BODIPY C_11_ (5 μM) was added to 20 × 10^6^ spermatozoa/mL, incubated for 30 min at 37 °C, and washed once (300 g for 7 min). At the end of this incubation time, 100 μL of sample were mixed with 2 μL H-42, (0.05 mg/mL in PBS) and 1.3 μL PI (0.5 mg/mL in PBS) and incubated for 10 min at 37 °C. Immediately before analysis by flow cytometry, 200 μL of PBS were added to each sample and the samples were mixed. The mean fluorescence intensity was expressed as arbitrary fluorescence units per 10^3^ viable spermatozoa.

### Experimental designs

#### Experiment 1: inter-boar, intra-boar and intra-ejaculate variability in SP-TAC

TAC-values were assessed in SP samples from 60 entire ejaculates (four ejaculates of 15 boars) to investigate variability among boars (inter-boar variability) and among ejaculates within the same boar (intra-boar variability). In addition, SP-TAC was measured in three different ejaculate portions from 43 ejaculates (one ejaculate per boar): the sperm-peak portion (first 10 mL) of sperm rich fraction (SRF); the rest of the SRF; and the post-SRF.

#### Experiment 2. Relationship between SP-TAC and sperm quality of semen stored samples

Two different experiments were performed to evaluate the putative influence of SP-TAC on the ability of boar sperm to tolerate preservation, either when stored as liquid extended semen (Exp. 2a) or following freezing and thawing (Exp. 2b). In Exp. 2a, a total of 90 semen samples from 90 ejaculates (one per boar) extended in commercial extender at 3 × 10^9^ sperm/mL to mimic conventional AI-semen doses, were stored at 15–17 °C during 72 h. Sperm quality was evaluated at 24 and 72 h of storage. A SP sample of each of the 90 ejaculates was used for measuring SP-TAC. In Exp. 2b, 14 ejaculates (7 boars, 2 ejaculates/boar) were cryopreserved in 3 separate portions: the first 10 mL of SRF; the rest of SRF; and a proportion mix of the first 10 mL of SRF, the rest of SRF and the post-SRF to simulate the entire ejaculate. The SP-TAC was measured in the first 10 mL of SRF, the rest of SRF and entire ejaculate samples of the 14 ejaculates. Sperm quality was evaluated at 30 and 150 min post-thawing.

#### Experiment 3: Relationship between SP-TAC and fertility post-AI

Fertility parameters, in terms of farrowing rate, litter size (total number of piglets born per litter) and fertility index (the total number of piglets born as a proportion of the number of sows inseminated), of 18 AI-boars were recorded over a 12-month period to evaluate the association between SP-TAC values and *in vivo* fertility. Weaned multiparous (1–7 farrows) Landrace and Large White sows housed in different farms were cervically inseminated (2–3 times per oestrus) using 24–72 h liquid stored semen doses (2,500 × 10^6^ sperm in 80 mL) elaborated from entire ejaculates. The number of inseminated sows per boar ranged between 107 and 801. The SP-TAC values were measured in three entire ejaculates per boar from those collected to elaborate the AI-semen doses (in order of include one SP-sample per boar per every 4 months, minimizing possible effects of seasonality).

### Statistical analysis

For data analysis, IBM SPSS Statistics 19.0 (IBM Spain, Madrid) was used. The residual data for each statistical variable were evaluated using the Kolmogorov-Smirnov test to check the assumption of normality, and those not normally distributed were arcsine- (data in percents) or log- (count data) transformed. In Exp.1, a mixed ANOVA including the effects of boar and ejaculate within boar was performed to investigate inter- and intra-boar variability on SP-TAC values, and intra-boar reliability was assessed by intra-class correlation [ICC (3,1)] in a two-way mixed approach. In Exp.2a, the Pearson parametric correlation test was used to measure the relationship between the SP-TAC values and the sperm quality and functionality, as evaluated at 24 and 72 h of storage at 15–17 °C. A hierarchical cluster analysis was carried out to identify naturally occurring groups within the SP-TAC data set, identifying three groups as high, moderate and low SP-TAC values. A one-way ANOVA was performed to verify the extent of the differences on SP-TAC values among the three groups. Then, the sperm quality and functionality data were considered in two ways as measured in each of the two storage times (absolute values) and as the difference in percentage between both storage times (relative values). A repeated-measures ANOVA was performed to evaluate the influence of SP-TAC group and storage time on absolute values, and a one-way ANOVA to evaluate the influence of SP-TAC group on relative values. In Exp. 2b, a mixed ANOVA model was performed to investigate differences on SP-TAC and sperm quality and functionality among P1, P2 and EE ejaculate portions, including boar and ejaculate within boar as random effects in the model. In Exp.3, to analyze the relationship between SP-TAC and boar fertility outcomes, the raw fertility dataset was corrected for parameters related to farm and sow by using the multivariate statistical model previously described by Broekhuijse *et al.*^17^. The Pearson parametric correlation test was used to measure the relationship between the SP-TAC values and fertility outcomes. A hierarchical cluster analysis was also carried out to identify naturally occurring groups within the SP-TAC data set of 18 boars, identifying two groups of boars showing high and low SP-TAC values. A one-way ANOVA was performed to investigate differences on fertility outcomes between the two groups of boars. The Bonferroni test was used for post-hoc analyses where appropriate. A value of *P* < 0.05 was accepted as the minimal level of significance. Data are shown as means ± standard error of the mean (SEM).

## Results

### Experiment 1: inter-boar, intra-boar and intra-ejaculate variability in SP-TAC

**Figure 1:**
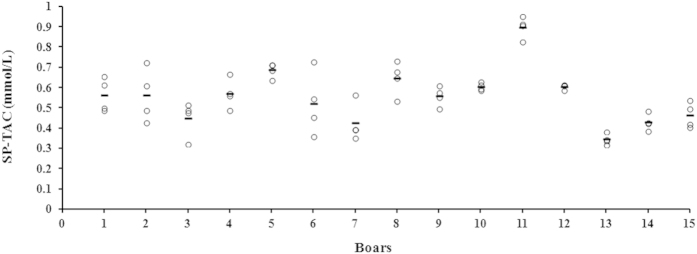
Scatterplot showing the seminal plasma total antioxidant capacity (SP-TAC) of ejaculates collected from15 boars (four ejaculates per boar). Circles show the SP-TAC value measured in each ejaculate and the lines show the mean for each boar.

The variability in SP-TAC values among boars (inter-boar variability) and between ejaculates within boar (intra-boar variability) was evaluated in 60 ejaculates collected from 15 boars (4 ejaculates per boar). Inter-boar variability was significant (*P* < 0.001) showing boar numbers 11 and 13 the most extreme SP-TAC values, with 0.96 ± 0.05 mmol/L and 0.31 ± 0.03 mmol/L, respectively (Fig. 1). Intra-boar variability was also significant (*P* < 0.001). Nevertheless, intra-boar variability was smaller than the inter-boar variability, as showed by an ICC (3,1) of 0.76 (CI: 0.56–0.90; 95%), indicating good reliability of SP-TAC measurements among ejaculates within a same boar.

**Figure 2:**
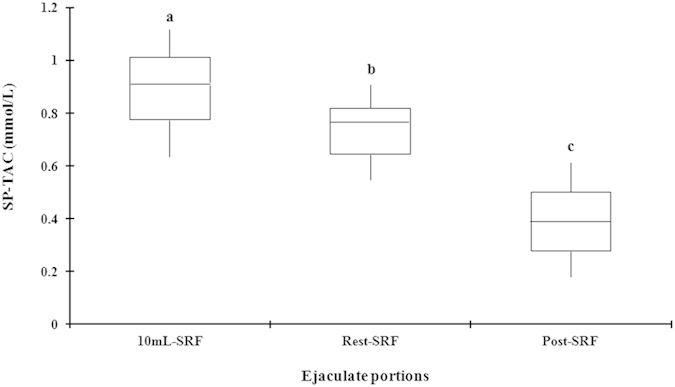
Box-whisker plot showing variation in seminal plasma total antioxidant capacity (SP-TAC) of the three ejaculate portions: the first 10 mL of spermatozoa-rich fraction (SRF), the rest of SRF and the post-SRF of 43 ejaculates (1 per boar). Boxes enclose the 25th and 75th percentiles; the line is the median; and the whiskers extend to the 5th and 95th percentiles. (a–c) indicate significant differences (*P* < 0.05) among the ejaculate portions.

The SP-TAC values in the different ejaculate-portions were assessed in 43 ejaculates (one per boar) with significant differences (*P* < 0.001) between the three fractions, showing the first 10 ml of SRF higher values (0.89 ± 0.02 mmol/L, range: 0.63–1.14) than the rest of SRF (0.72 ± 0.02 mmol/L, range: 0.53–1.04), which in turn were higher than those of post-SRF (0.41 ± 0.02 mmol/L, range: 0.12–0.62) (Fig. 2).

### Experiment 2. Relationship between SP-TAC and sperm preservation

**Figure 3:**
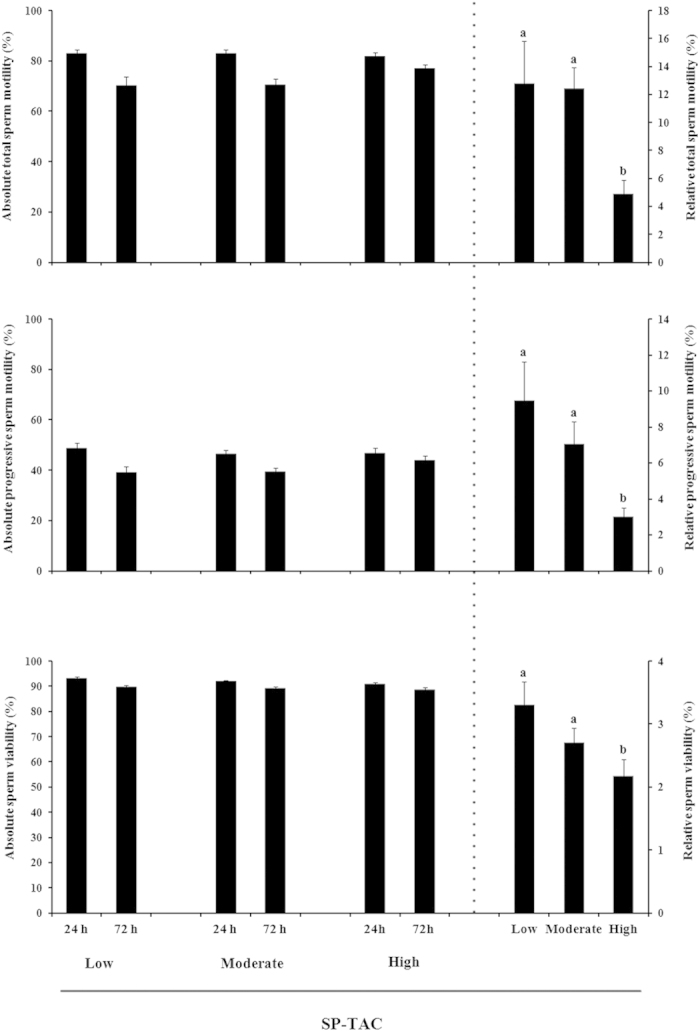
Histograms showing sperm quality parameters measured of artificial insemination semen doses stored 72 h of storage at 15–17 °C and hierarchically grouped according to the seminal plasma total antioxidant capacity (SP-TAC) as low (from 0.03 to 0.45 mmol/L, n° = 21), moderate (from 0.47 to 0.67 mmol/L, n° = 40) and high (from 0.68 to 1.04 mmol/L, n° = 29). Data are showed as measurements recorded at 24 h and 72 h of storage (absolute values, to the left of each chart) and as the difference in percentage between both storage times (relative values, to the right of each chart). (a,b) indicate significant differences (*P* < 0.05) among different SP-TAC groups.

In Exp. 2a, the relationship between SP-TAC values and sperm quality and functionality was assessed in liquid stored semen AI-samples from 90 ejaculates. The SP-TAC values varied widely among ejaculates, ranging from 0.03 mmol/L to 1.04 mmol/L. The SP-TAC did not show significant correlations with any of the sperm quality and functionality parameters evaluated. Given this, the ejaculates were classified (hierarchical clustering) into 3 groups as with high (from 0.68 to 1.04 mmol/L, n° = 29), moderate (from 0.47 to 0.67 mmol/L, n° = 40) or low (from 0.03 to 0.45 mmol/L, n° = 21) SP-TAC (*P* < 0.001). The 90 extended semen samples were stored at 15–17 °C and sperm quality and functionality evaluated at 24 and 72 h of storage. The total motility, progressive motility and sperm viability did not differ among SP-TAC groups, neither at 24 nor at 72 h of storage (Fig. 3). The sperm quality decreased (P < 0.001) across storage time, irrespective of SP-TAC group, albeit total motility, progressive motility and sperm viability remained above 70%, 35% and 80%, respectively, after 72 h of storage in the three SP-TAC groups. Despite this, the relative decline (difference in percentage between the two storage times) was larger (*P* < 0.05) in semen samples from ejaculates with low SP-TAC than those with high SP-TAC (Fig. 3). Basal intracellular H_2_O_2_ generation in viable spermatozoa did not differ among SP-TAC groups and it was higher by 24 h (mean arbitrary fluorescence intensity was 9.78 ± 0.54 FU × 10^6^ viable sperm) than at 72 h (7.36 ± 0.29 FU  × 10^6^ viable sperm) of storage. Similarly, the percentage of viable spermatozoa showing lipid peroxidation did not differ between SP-TAC groups, and neither differed between the two evaluation times (mean arbitrary fluorescence intensity was 8.57 ± 1.19 and 11.91 ± 2.02 × 10^3^ viable sperm at 24 and 72 h of storage, respectively).

**Figure 4:**
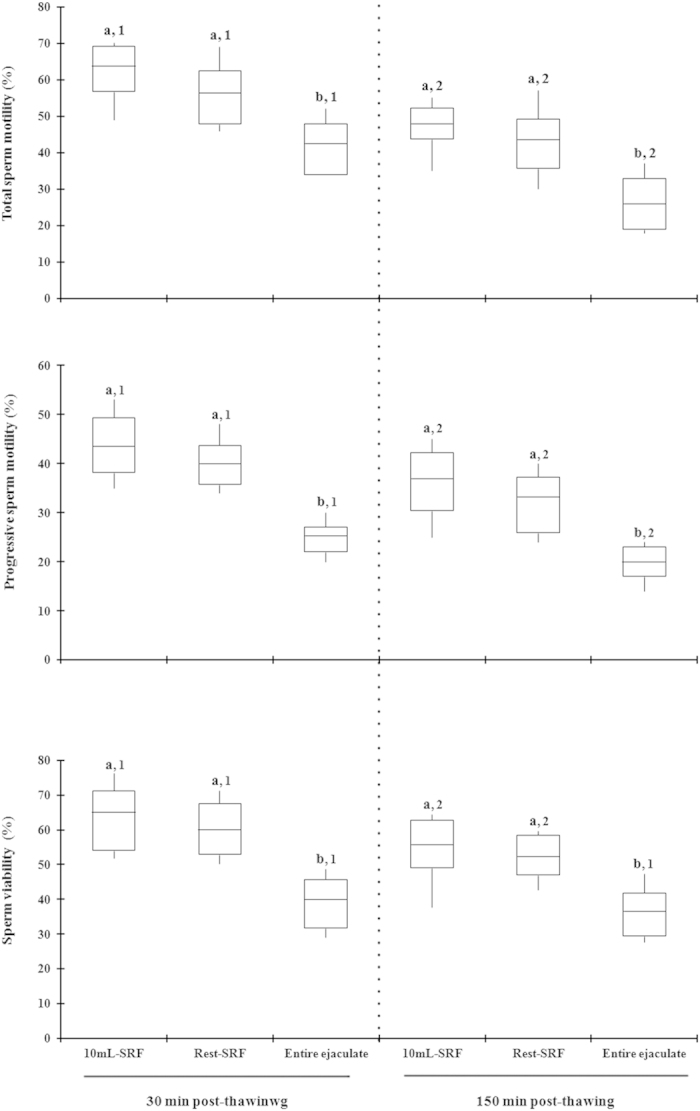
Box-whisker plot showing variations in sperm quality parameters assessed at 30 (left) and 150 (right) min post-thawing in cryopreserved sperm samples of first 10 mL of sperm rich ejaculate fraction (10 ml-SRF), the rest of SRF or a reconstituted entire ejaculate of 14 ejaculates (2 per boar). Boxes enclose the 25th and 75th percentiles; the line is the median; and the whiskers extend to the 5th and 95th percentiles. a,b indicate significant differences (*P* < 0.05) among the ejaculate portions within the same post-thaw incubation time and 1,2 indicate significant differences (*P* < 0.05) between post-thaw incubation times within the same ejaculate portion.

**Figure 5:**
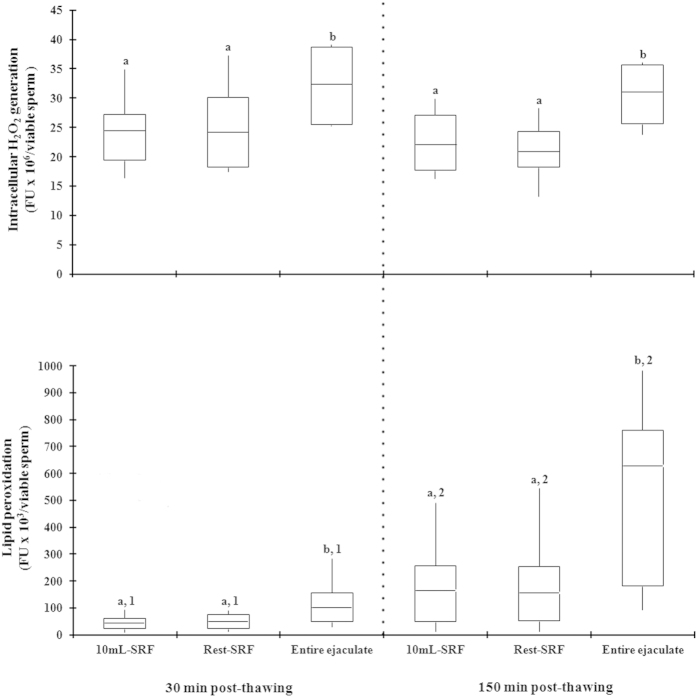
Box-whisker plot showing variations in sperm functionality parameters assessed at 30 (left) and 150 (right) min post-thawing in cryopreserved sperm samples of first 10 mL of sperm rich ejaculate fraction (P1), the rest of sperm rich ejaculate fraction (P2) or a reconstituted entire ejaculate (EE, mixing aliquots of the portions collected separately) of 14 ejaculates (2 per boar). Boxes enclose the 25th and 75th percentiles; the line is the median; and the whiskers extend to the 5th and 95th percentiles. a,b indicate significant differences (*P* < 0.05) among the ejaculate portions within the same post-thaw incubation time and 1,2 indicate significant differences (*P* < 0.05) between post-thaw incubation times within the same ejaculate portion. FU: arbitrary fluorescence units.

In Exp. 2b, the relationship between SP-TAC values and sperm quality and functionality were assessed in frozen-thawed semen samples of three different ejaculate portions collected from 14 ejaculates. The SP-TAC values differed (*P* < 0.001) between ejaculate-portions, showing the first 10 mL of SRF the highest and the post-SRF the lowest values. Post-thaw total- and progressive sperm motility and viability were similar in the first 10 mL of SRF and the rest of SRF yet higher (*P* < 0.01) than in the entire ejaculate (mainly post-SRF), irrespective of post-thaw evaluation time. The post-thaw sperm quality decreased (P < 0.001) across incubation time, irrespective of ejaculate fraction (Fig. 4). Basal intracellular H_2_O_2_ generation and lipid peroxidation in thawed viable spermatozoa was higher (*P* < 0.01) in the entire ejaculate (mainly post-SRF) than in the first 10 mL of SRF or rest of SRF (Fig. 5).

### Experiment 3: Relationship between SP-TAC and fertility post-AI

Fertility outcomes of the 18 boars are summarized in Table 1. The SP-TAC values were positively correlated with litter size (*r* = 0.54, *P* < 0.05) and fertility index (*r* = 0.52, *P* < 0.05). The 18 boars were classified (hierarchical clustering) into 2 groups as having high (from 0.61 to 0.91 mmol/L, n° = 10) or low (from 0.29 to 0.52 mmol/L, n° = 8) mean SP-TAC values (*P* < 0.001). Boars with high SP-TAC values showed averaged higher fertility outcomes than those with low SP-TAC values, as evidenced in farrowing rates (87.72 ± 0.85 *vs* 84.49 ± 0.80, *P* < 0.05), litter sizes (13.57 ± 0.15 *vs* 12.64 ± 0.19, *P* < 0.01) and fertility index (11.91 ± 0.21 *vs* 10.68 ± 0.21, *P* < 0.01).

## Discussion

The results of the first experiment clearly showed that SP-TAC varies significantly among boars as well as, albeit at a lower level, among ejaculates from the same boar. This variability, in agreement to that recently observed in bulls^18^, is not surprising as the concentration of specific antioxidants varies among boars and ejaculates^19^,^20^. As the SP-TAC is important to keep extracellular ROS production within affordable levels for sperm functionality, it is reasonable to think that spermatozoa from ejaculates with low SP-TAC may have a compromised functional lifespan.

The experiment designed to evaluate an eventual relation between SP-TAC and sperm quality and functionality in liquid semen extended samples was performed using designed commercial AI-doses stored at 15–17 °C for 72 h. This storage time was chosen because it is the most commonly used in commercial swine AI programs, owing to the probability of decrease in fertility of older AI-doses^21^. The lack of correlation between SP-TAC and sperm quality and functionality found in the 90 AI-semen samples evaluated was noticeable, despite the extent of range in SP-TAC values. These results contrast with growing evidence, mainly in humans, indicating a positive relationship between SP-TAC and sperm quality and functionality^22^,^23^. In general, these studies indicated that semen samples with low SP-TAC generate excessive amounts of ROS leading to lipid peroxidation and subsequent impairment of sperm quality and functionality. In the present study, the percentages of viable and motile spermatozoa were highest in two evaluation times, 24 and 72 h of storage, not surprising considering the boars used are highly selected breeding males for AI. In addition, the basal intracellular ROS generated in viable sperm (measured as H_2_O_2_, the major free radical mediating direct ROS effects in boar sperm^24^) were very low and the subsequent lipid peroxidation practically unmeasurable, regardless of the SP-TAC values of semen samples. These results, consistent with those of Guthrie and Welch^9^ and Waberski *et al.*^21^, would indicate that the studied semen samples were stored under optimal conditions, maintaining enough endogenous ROS defences to keep ROS generation in viable spermatozoa within physiological safe range. Then, under these circumstances it would be reasonable to assume that the measurement of SP-TAC to foresee sperm quality and functionality would be less relevant. However, when evaluating the decline in sperm quality between 24 and 72 h, it was noticed that semen samples grouped as of low SP-TAC showed a slight but significantly greater decline in motility and viability compared to semen samples grouped as with high SP-TAC. These results highlighted that SP-TAC would protect boar spermatozoa during liquid storage, particularly if semen samples were stored under suboptimal conditions or provided by boars whose spermatozoa were more sensitive to liquid storage.

Variability in sperm freezability among boars is a known issue for years^25^ remaining still valid today^4^. Recently, Vilagran *et al.*^7^ demonstrated that quantitative differences in SP-composition play an important role in such variability, as Glutathione Peroxidase 5 and Fibronectin-1 were higher in SP of ejaculates from boars with good- than those with bad sperm freezability. In addition to inter-boar variability, Peña *et al.*^26^ and other researchers later^27^,^14^ showed that sperm freezability also varied between portions of the same ejaculate, spermatozoa from the first 10 mL of SRF depicting better freezability than those from the rest of the ejaculate. The results of the present study are in agreement with the above studies but also showed, as novelty, that differences in SP-TAC values among ejaculate portions could explain, at least in part, this peculiar intra-ejaculate variability in boar sperm freezability. The ejaculate portions with better or worse sperm cryosurvival were clearly related to those with higher or lower SP-TAC values, respectively. In contrast to liquid stored semen, cryopreservation is highly stressful to boar spermatozoa^28^. SP-TAC was able to protect boar spermatozoa against cryodamage; as clearly evidenced by the lower ROS generation and lipid peroxidation shown by thawed viable sperm from ejaculate portions with highest SP-TAC values. These results would suggest that semen samples with high values of SP-TAC undergo less oxidative stress during cryopreservation, resulting in better sperm cryosurvival, measured in terms of viable, motile spermatozoa with intact membranes, post-thaw. The protective effect of SP-TAC would not happen *in situ* during the freezing and thawing processes, because most SP is routinely removed before freezing in order to concentrate the spermatozoa in a minimum volume^14^. Then, the protective effect of SP-TAC would happen either as adsorbed elements or during the elapsed time between ejaculations and freezing, when spermatozoa remained surrounded by their own SP. This elapsed time is usually between 16 and 24 h^29^, as used in our experiment. Probably it is during this long incubation period before freezing that some antioxidant compounds within the SP-TAC, particularly those lipid-soluble, incorporate into sperm membranes making boar spermatozoa more resilient to freezing and thawing stresses, as documented in turkey spermatozoa^30^.

The most relevant finding of the present study is the positive correlation between the SP-TAC values and AI-fertility outcomes of boars. Data are from over 5,000 sows inseminated with semen AI-doses of 18 boars, which give considerable strength to the findings. Sows were inseminated with liquid semen AI-doses stored at 15–17 °C for 24–72 h, like those used in the second experiment. Interestingly, in this particular experiment, the SP-TAC was not correlated with sperm quality or functionality neither at 24 h nor 72 h of storage. Sperm quality, in terms of viability and motility, was good at both storage times and in all the semen AI-doses evaluated, and we only noticed a slightly more pronounced decline in sperm quality in the AI-semen doses with less SP-TAC from 24 h until 72 h of storage. The good sperm quality and functionality of liquid stored semen AI-doses would agree with the overall high fertility outcomes shown by all 18 boars. Despite this, the slight differences in fertility outcomes among boars show a direct relationship with SP-TAC values, highlighting that SP-TAC present in the AI-semen doses would contribute to improved fertilizing capacity and subsequent embryo development. The relationship between some specific SP-antioxidants and fertility outcomes in swine recently proved by Novak *et al.*^6^ and Barranco *et al.*^31^ would support these results. Although the present study does not disclose how SP-TAC contributes to fertility outcome, all together the results of the three experiments allow elaborating some hypotheses. The first one would be that some SP-antioxidants bind to the sperm membranes making sperm more resistant and long-lived in their transit through the genital tract of the sow. The second one, not excluding the first one, would be that SP-antioxidants help to prevent or at least minimize the damaging effects that exogenous ROS, generated by leukocytes present in the uterus, would have on the AI-spermatozoa. In this sense, it is well known that once inseminated and into the uterus, spermatozoa must face new ROS-attacks^32^ and that antioxidants present in the SP that go along with the spermatozoa during AI are involved in counteracting the detrimental effects of ROS^11^,^33^.

In conclusion, the results showed that SP-TAC differs among boars and, to a lesser degree, among ejaculates within boar. In addition, SP-TAC also differs among ejaculate portions, showing the first 10 mL of the SRF the highest SP-TAC values. The study also evidences that SP-TAC contributes to the ability of boar spermatozoa to better sustain preservation, particularly cryopreservation. Finally, the most commercially relevant finding of this study was that boars with higher SP-TAC levels generally have better fertility outcomes, suggesting that SP-TAC should be considered as a potential fertility biomarker for AI-boars.

## Additional Information

**How to cite this article**: Barranco, I. *et al.* High total antioxidant capacity of the porcine seminal plasma (SP-TAC) relates to sperm survival and fertility. *Sci. Rep.* **5**, 18538; doi: 10.1038/srep18538 (2015).

## Figures and Tables

**Table 1:** Fertility AI-outcomes and seminal plasma total antioxidant capacity (SP-TAC, mmol/L) of the liquid semen of 18 boars (2,500 × 10^6^ sperm/dose).

Boars	SP-TAC (mean ± SEM)	Sows inseminated (n°)	Farrowing Rate (%)	Total Piglets Born (mean ± SEM)	Fertility Index^a^
1	0.91 ± 0.04	225	84.00 ± 2.68	13.50 ± 0.28	11.34
2	0.90 ± 0.01	382	86.65 ± 1.70	12.98 ± 0.16	11.25
3	0.77 ± 0.06	456	84.43 ± 1.88	12.79 ± 0.16	10.80
4	0.72 ± 0.01	801	86.64 ± 1.29	13.92 ± 0.12	12.06
5	0.71 ± 0.01	294	85.71 ± 2.25	13.91 ± 0.24	11.92
6	0.67 ± 0.04	546	89.01 ± 1.47	13.96 ± 0.14	12.43
7	0.66 ± 0.05	163	90.18 ± 1.99	14.06 ± 0.27	12.68
8	0.66 ± 0.02	350	90.86 ± 2.75	13.87 ± 0.15	12.60
9	0.66 ± 0.02	487	91.79 ± 1.50	13.74 ± 0.16	12.61
10	0.61 ± 0.03	579	87.91 ± 1.57	12.94 ± 0.15	11.38
11	0.52 ± 0.04	111	88.29 ± 3.64	12.44 ± 0.32	10.98
12	0.52 ± 0.02	107	84.11 ± 4.53	12.64 ± 0.30	10.64
13	0.51 ± 0.02	140	87.86 ± 3.45	13.27 ± 0.29	11.66
14	0.46 ± 0.01	182	83.52 ± 3.00	12.48 ± 0.31	10.42
15	0.44 ± 0.06	131	83.21 ± 2.80	11.80 ± 0.29	9.82
16	0.44 ± 0.01	191	83.25 ± 3.47	13.13 ± 0.22	10.93
17	0.34 ± 0.02	189	83.07 ± 2.78	13.21 ± 0.24	10.97
18	0.29 ± 0.02	212	82.60 ± 3.19	12.06 ± 0.36	10.05

Boars were hierarchically grouped as high (1-10) and low (11-18) SP-TAC values.

^a^The total number of piglets born as a proportion of the number of sows inseminated by each boar.
